# *Drechslerella stenobrocha* genome illustrates the mechanism of constricting rings and the origin of nematode predation in fungi

**DOI:** 10.1186/1471-2164-15-114

**Published:** 2014-02-08

**Authors:** Keke Liu, Weiwei Zhang, Yiling Lai, Meichun Xiang, Xiuna Wang, Xinyu Zhang, Xingzhong Liu

**Affiliations:** 1 State Key Laboratory of Mycology, Institute of Microbiology, Chinese Academy of Sciences, No 3 1st Beichen West Rd., Chaoyang District, Beijing 100101, China; 2 University of Chinese Academy of Sciences, Beijing 100039, China

**Keywords:** Nematode-trapping fungi, Comparative genomic analysis, Origin of nematode predation, Transcriptomes, Trapping mechanism

## Abstract

**Background:**

Nematode-trapping fungi are a unique group of organisms that can capture nematodes using sophisticated trapping structures. The genome of *Drechslerella stenobrocha*, a constricting-ring-forming fungus, has been sequenced and reported, and provided new insights into the evolutionary origins of nematode predation in fungi, the trapping mechanisms, and the dual lifestyles of saprophagy and predation.

**Results:**

The genome of the fungus *Drechslerella stenobrocha*, which mechanically traps nematodes using a constricting ring, was sequenced. The genome was 29.02 Mb in size and was found rare instances of transposons and repeat induced point mutations, than that of *Arthrobotrys oligospora*. The functional proteins involved in nematode-infection, such as chitinases, subtilisins, and adhesive proteins, underwent a significant expansion in the *A. oligospora* genome, while there were fewer lectin genes that mediate fungus-nematode recognition in the *D. stenobrocha* genome. The carbohydrate-degrading enzyme catalogs in both species were similar to those of efficient cellulolytic fungi, suggesting a saprophytic origin of nematode-trapping fungi. In *D. stenobrocha*, the down-regulation of saprophytic enzyme genes and the up-regulation of infection-related genes during the capture of nematodes indicated a transition between dual life strategies of saprophagy and predation. The transcriptional profiles also indicated that trap formation was related to the protein kinase C (PKC) signal pathway and regulated by Zn(2)–C6 type transcription factors.

**Conclusions:**

The genome of *D. stenobrocha* provides support for the hypothesis that nematode trapping fungi evolved from saprophytic fungi in a high carbon and low nitrogen environment. It reveals the transition between saprophagy and predation of these fungi and also proves new insights into the mechanisms of mechanical trapping.

## Background

Predation is one fungal life strategy to manipulate free-living nematode dynamics in nature and capture nitrogen [1-3]. Fungi have evolved sophisticated trapping structures, including constricting rings traps [1,4] and five types of adhesive traps (sessile adhesive knobs, stalked adhesive knobs, adhesive nets, adhesive columns, and non-constricting rings) [5,6], with which they capture nematodes for nutritional purpose [7,8]. Based on molecular clock and fossil evidence, nematode predatory ascomycetes were estimated to have originated as a result of mass extinctions in the Permian and Triassic [9,10]. The hypothesis holds that dead creatures caused by mass extinctions were rich in carbon but poor in nitrogen, so direct capture of nitrogen rich living animals would give predatory fungi a competitive advantage over strictly saprophytic fungi [11]. Constricting rings is considered to be the ancestral strategy after which all of the adhesive traps evolved [6].

Most nematode-trapping fungi can live both saprophytically on organic matter and as predators by capturing tiny animals [12]. Abundant nematode-trapping fungi have been detected in niches that that are rich in decayed organic matter, such as decayed leaves and wood [13]. Nematode-trapping fungi that form constricting rings have high density in the soil with rich organic matters whereas the fungi that form adhesive nets mostly diversity in the relatively poorer soil [14]. They are also influenced by the population density of nematodes, which are key nutritional resources for these fungal populations [15].

**Figure 1:**
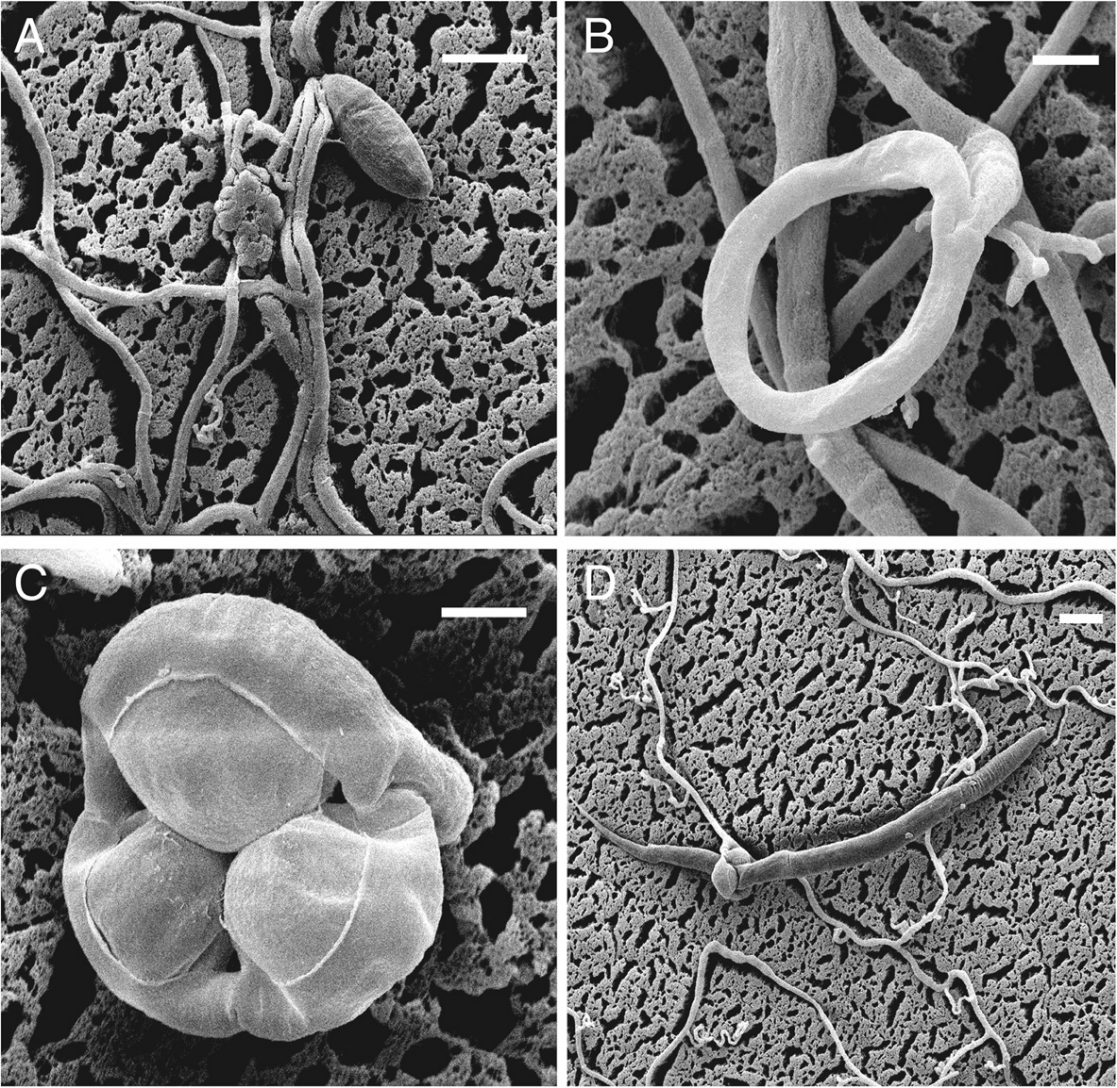
**Characteristics of *D. stenobrocha *used in the sequenced nematode-trapping fungus, showing the amazing mechanical trapping process.** The trapping process includes ring formation and ring constriction. **A)** The free living hyphae and spore; **B)** Ring without constriction; **C)** Ring induced to constrict; **D)** Nematode is capturing. Scale bar: **A**, **D** = 20 μm; **B**, **C** = 5 μm.

Traps are usually produced from hyphae in the presence of nematodes [4] or are induced by peptides, or nematode extracts from the nematodes [16]. *Drechslerella stenobrocha* (Ascomycota: Orbiliaceae) is a nematode-trapping species that forms constricting rings, the most sophisticated trapping structure, that consist of three ring cells capturing nematodes actively (Figure 1A, B) [17]. When a nematode enters the ring and contacts the inner surfaces of the ring cells, G protein-coupled receptors activate a down-stream signal pathway that includes cyclic adenosine monophosphate (cAMP), inositol-1, 4, 5-triphosphate (IP3), and Ca^2+^[18]. Subsequently, the ring cells rapidly (within 0.1 s) triple their volume and firmly lasso the nematode (Figure 1C, D) [19].

*Arthrobotrys oligospora* (Ascomycota: Orbiliaceae) is an adhesive network-forming nematode-trapping fungus and a model organism for understanding the interactions between fungi and nematodes [20]. It can also parasitize other fungi and colonize plant roots [5,16,21]. Its genome sequence, the first available for a nematode-trapping fungus, provided information about some of the proteins responsible for trap formation, including G-protein coupled receptors, adhesive proteins, cell division cycle (CDC37), peroxisome-related proteins, and proteins involved in energy supplementation [22]. Although other fungal genomes, such as those of entomopathogenic fungi, have identified enzymes like subtilisins and chitinases to be important virulence factors in host barrier degradation [23,24], the available fungal genome data are not sufficient to elucidate the origin of fungal predation to nematodes or the trapping mechanisms. Since *D. stenobrocha* uses a constricting-ring mechanical trapping mechanism, the comparison of its genome with that of *A. oligospora* should understand the origin of nematode predation and the mechanisms.

To test the hypothesis that trapping fungi originated in carbon-rich but nitrogen-poor niches as well as to understand the nematode predatory lifestyle and constricting-ring trapping mechanism, the genome of *D. stenobrocha* was analyzed and compared with that of the network-forming fungus *A. oligospora* as well as those of non-trapping fungi. The results provide a comprehensive understanding of the biology of nematode-trapping fungi and crucial data for further studies of their trapping mechanisms.

## Results

### The *D. stenobrocha* genome is smaller than that of the phylogenetically closely related *A. oligospora*

**Figure 2:**
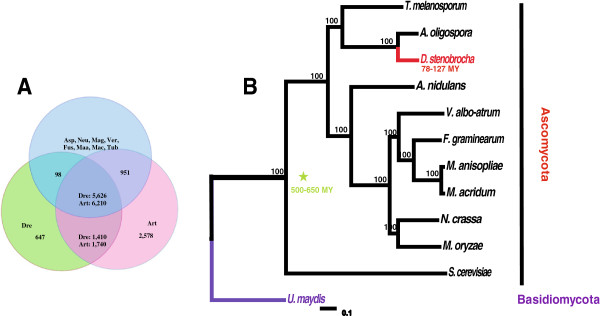
**Homology and phylogenic relationships of *D. stenobrocha *and other selected fungi. A)** Predicted proteins in *Drechslerella stenobrocha* (Dre) and *Arthrobotrys oligospora* (Art) were compared with genome coding genes in *Aspergillus nidulans* (Asp), *Neurospora crassa* (Neu), *Magnaporthe oryzae* (Mag), *Verticillium albo-atrum* (Ver), *Fusarium graminearum* (Fus), *Metarhizium anisopliae* (Maa), *Metarhizium acridum* (Mac) and *Tuber melanosporum* (Tub). **B)** The phylogeny tree was constructed using RAxML. Scale bar represents the number of substitutions per site. MY = million years (DRYAD accession: doi:10.5061/dryad.9n6q6; http://datadryad.org).

The 29.02-Mb genome of *D. stenobrocha* was sequenced with Solexa (~80× coverage), with the NCBI accession of ASQI01000000. It yielded 142 scaffolds, 134 of which were 2-kb or larger, and had a repeat content of 0.97% (Table 1). In contrast, genome of *A. oligospora* was 40.07 Mb in size, with 323 scaffolds, 252 of which were 2-Kb or larger [22]. A total of 11,479 genes were predicted for *A. oligospora*, whereas only 7,781 genes, with few duplicates, were estimated in the genome of *D. stenobrocha* (Additional file 1: Figure S1, Additional file 2: Table 1). The two genomes shared 7,036 homologs with more than 80% predicted amino-acid similarity, covering more than 90% of the genes in the *D. stenobrocha* genome. However, this number dropped to 5,626–6,210 when comparing the *D. stenobrocha* or *A. oligospora* genomes with those of other selected species that include two entomopathogens (*Metarhizium acridum* and *Metarhizium anisopliae*), three phytopathogens (*Magnaporthe oryzae*, *Verticillium albo-atrum* and *Fusarium graminearum*), two saprobes (*Aspergillus nidulans* and *Neurospora crassa*), and one symbiotic fungus (*Tuber melanosporum*) (Figure 2A, Additional file 2: Table S1). More than 1,400 predicted genes matched strictly between *D. stenobrocha* and *A. oligospora*, indicating their close phylogenetic relationship. When *D. stenobrocha* was compared with other selected fungi, only 647 orphan sequences (without known homologs) were predicted in its genome, while the *A. oligospora* genome had 2,578 orphan sequences.

**Table 1:** **Main features of the *Drechslerella stenobrocha *** **genome**

**Features**	***D. stenobrocha***
Assembled size (Mb)	29.02
Scaffolds (larger than 2 Kb)	134
Scaffolds (total)	142
Scaffold N50 (bp)	434,454
Coverage (fold)	80 × (Solexa)
G + C content (%)	52.5
Repeat rate (%)	0.92
Coding rate (%)	41.55
protein-coding genes	7,781
Gene density (genes per Mb)	268.3
GC exonic (%)	55.31
GC intronic (%)	49.24
Exons per gene	3.57
tRNA genes	82

Phylogenetic analyses of *D. stenobrocha*, *A. oligospora*, and 10 other fungal species (Additional file 2: Table S1) also confirmed that the former two were much more closely related to one another than to the other sampled species. *Drechslerella stenobrocha* diverged 78–127 million years (MY) ago, earlier than the insect parasites *Metarhizium anisopliae* and *Metarhizium acridum* (26–34 MY), but later than the plant parasite *Fusarium graminearum* (200–260 MY) and the saprophyte *Neurospora crassa* (240–300 MY) (Figure 2B).

### Fewer repeat-induced point mutations and transposons in the *D. stenobrocha* genome

A lack of transposases (1 in *D. stenobrocha* versus 11 in *A. oligospora*) limited the occurrence of transposons (one transposon of 984 bp) in the *D. stenobrocha* genome, resulting in its smaller genome size. Like the fungi such as *Verticillium dahliae* and *Verticillium albo-atrum*[25], more than 95% of the repetitive sequences (1.42% transposable elements) in the *A. oligospora* genome occurred in non-syntenic regions. The 256 transposable elements in the *A. oligospora* genome were classified into different repeat families. Class I retrotransposons comprised 50% of the repetitive fraction, unclassified transposable elements comprise almost all of the remainder.

Repeat-induced point (RIP) mutations, referred to as CpG to TpA mutations in duplicated sequences, are a defense mechanism to suppress the activity of transposases in fungal genomes. They were first described in the genome of *Neurospora crassa*[26,27]. The gene NCU02034 (http://www.ncbi.nlm.nih.gov/protein/AAM27408.1) in *N. crassa*, which is only known to be required for RIP, lack its homologous gene found in the *D. stenobrocha* genome [27]. In addition, RIPs occur only in repetitive regions larger than 500 Kb and no such repetitive sequences were found in *D. stenobrocha*. These indicate the lack of RIPs. However, significant evidence of RIPs was found in the *A. oligospora* genome, especially in the repeat-rich non-syntenic regions, which might explain the arising of numerous orphan genes related to nematode-infection.

### Genes enriched in non-syntenic, repeat-rich regions are associated with fungal predation

**Figure 3:**
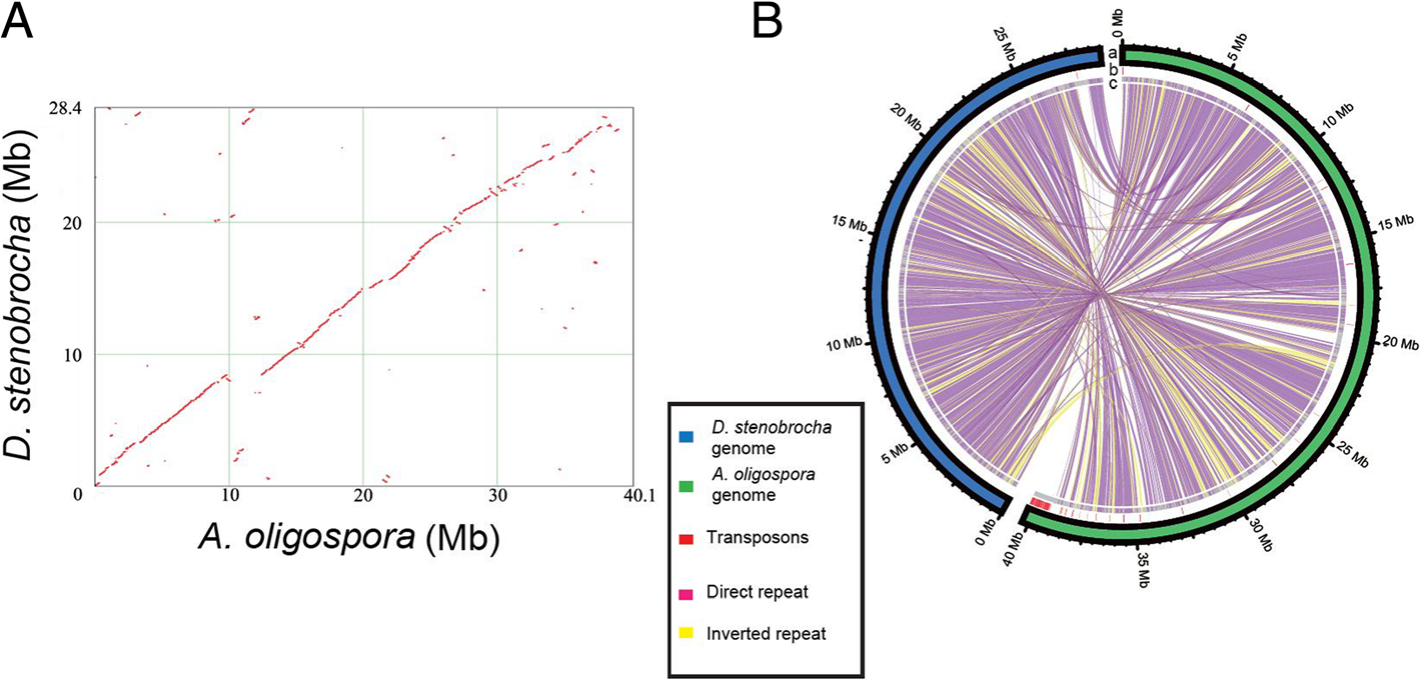
**Genomic relationship between *D. stenobrocha *and *A. oligospora*. A)** Syntenic relationship between *A. oligospora* and *D. stenobrocha*; **B)** Global view of syntenic alignments between *D. stenobrocha* (blue) and *A. oligospora* (green) and the distribution of transposable elements (TE). a: Genome sizes of the two genomes; b: Diversity of TE, red lines refer to TE; c: Grey part in the inner cycle refers to the non collinear region in the genomes; lines in the cycle indicate the syntenic region of the two genomes.

The repeat-rich but gene-poor regions were hypothesized to contain genes associated with host adaptations based on genome analyses of phytopathogenic fungi such as *Phytophthora* species and *Leptosphaeria maculans*[28,29]. Similar results were obtained for nematode-trapping fungi. The syntenic regions of 29 Mb (Figure 3A) contained more than 80% of the predicted genes in *A. oligospora*, whereas over 99% of the predicted genes in *D. stenobrocha* genome were located in the highly syntenic regions. Most of the predicted homologous genes were highly conserved between those two genomes. Compared with the *D. stenobrocha* genes in the syntenic regions, fewer than 50% of the genes in the non-syntenic regions had annotations in the Pfam database [30], indicating that the genes in non-syntenic regions have unique gene functions. These genes may have originated because of the rich repetitive sequences and RIPs in the non-syntenic regions. Compared to the syntenic regions, 256 of the 260 transposable elements in the *A. oligospora* genome occurred in the non-syntenic regions (Figure 3B), and RIPs were much more frequent in the coding regions of non-syntenic sequences. A large number of genes associated with nematode trapping, including those encoding lectins, subtilisins, chitinases, and adhesive proteins, were found in the less-syntenic and repeat-rich regions in the *A. oligospora* genome [22].

### Pathogenic functional proteins associated with nematode-predation in *D. stenobrocha* and *A. oligospora*

**Figure 4:**
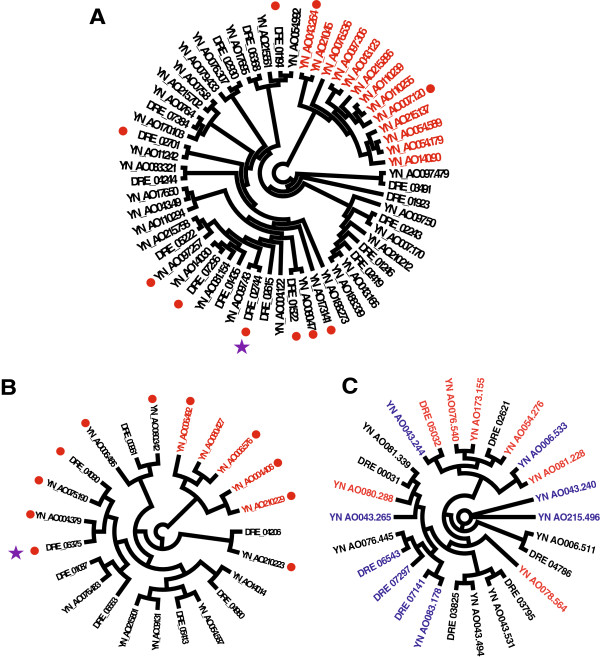
**Unrooted phylogenetic trees showing differences in gene expansion in *D. stenobrocha *and *A. oligospora. *A)** Subtilisins (peptidase S08); **B)** Chitinases (GH18); **C)** Lectins. Red branches in file **A** and **B** identify genes only present in *A. oligospora* genome. Red branches in file **C** identify the fungal-fucose lectin coding genes and blue branches identify GalNAc-type lectin coding genes. Secreted enzymes are marked with red circles and differently expressed genes during nematode-trapping are marked with purple stars.

Chitinases and subtilisins are crucial pathogenic factors for fungal infection of both nematodes and insects. In entomopathogenic fungi such as *Cordyceps militaris*, *M. anisopliae*, and *M. acridum*, these enzymes can degrade insect cuticles [23,24]. The glycoside hydrolase 18 (GH18) family of chitinases restrain the growth of first-stage juveniles of the nematode *Meloidogyne hapla* and appears to be involved in cuticle degradation during nematode infection [31,32]. A total of 17 genes encoding subtilisins were found in the *D. stenobrocha* genome, fewer than in the other genomes examined (Table 2). In contrast, the relatively high number (40) of subtilisin genes in the *A. oligospora* genome suggested an enhanced capacity of nematode infection. Thirteen of them were located in non-syntenic regions and shared low amino acid similarity with their homologs in the *D. stenobrocha* genome (Figure 4A). Likewise, there were eight GH18 chitinase genes in the *D. stenobrocha* genome but 15 in the *A. oligospora* genome; five of those genes in the *A. oligospora* genome were located in non-syntenic regions and shared low amino acid similarity with their homologs in the *D. stenobrocha* genome (Figure 4B). Considering *A. oligospora* as a fungal pathogen, these genes may also enable *A. oligospora* to digest the fungal cell wall and to be pathogenic to a fungus [22].

**Table 2:** **Number of genes involved in chitin, cellulose and protein degradation of *D. stenobrocha *** **and selected fungi**

	**Chitinase**	**Cellulose degradation**						**Protein degradation**	
**Protein families**	**GH18**	**GH5**	**GH6**	**GH7**	**GH74**	**GH61**	**CBM1**	**A01**	**S08**
*D. stenobrocha*^a^	8	15	1	4	3	12	36	19	17
*A. oligospora*	15	21	2	6	4	26	84	30	42
*M. acridum*	19	9	0	0	1	1	2	24	31
*M. anisopliae*	27	9	0	0	1	2	4	32	45
*T. melanosporum*	5	7	0	0	1	3	3	3	6
*A. nidulans*	17	16	2	3	3	9	6	10	2
*N. crassa*	12	6	3	5	2	16	21	18	6
*M. oryzae*	15	13	3	6	2	23	19	20	24
*V. albo-atrum*	13	13	4	6	4	21	22	16	20
*F. graminearum*	18	14	1	2	2	14	12	18	26

The annotation is based on CAZy classification (http://www.cazy.org) and MEROPS database (http://merops.sanger.ac.uk). The subfamilies are listed by the functions it involved in.

^a^Abbreviation for the fungal genera: *Drechslerella stenobrocha*, *Arthrobotrys oligospora*, *Metarhizium acridum*, *Metarhizium anisopliae*, *Tuber melanosporum*, *Aspergillus nidulans*, *Neurospora crassa*, *Magnaporthe oryzae*, *Verticillium albo-atrum*, *Fusarium graminearum*.

Pathogenic fungi can recognize and attach to their hosts via adhesive proteins [33,34]. Extracellular proteins from plant- and animal-pathogenic fungi that contain an eight-cysteine CFEM domain have been predicted to be involved in signal transduction or adhesion in plant host–pathogen interactions [24,35]. Genomic sequences revealed that there were also large numbers of CFEM-containing proteins in nematode-trapping fungi (12 in *D. stenobrocha*, 17 in *A. oligospora*), similar to insect pathogens (17 in *M. anisopliae* and 11 in *M. acridum*). The proteins contain a signal for secretion were presented much better in nematode-trapping fungi (11 in *D. stenobrocha*, 12 in *A. oligospora*) than in entomopathogens (4 in *M. anisopliae*, 2 in *M. acridum*) (Additional file 2: Table S2) [24], indicating that these genes may function in nematode trapping. Other putative adhesive proteins, such as those containing the GLEYA domain, which binds the lectin-like ligand domain [36], have also been implicated in nematode trapping. Six genes encoding GLEYA-containing proteins were identified in each nematode-trapping species (versus an average 4.5 in the entomopathogens; Additional file 2: Table S2). These adhesive proteins may play more important roles in hydrophilic soil, compared the hydrophobic phyloplane environment of entomopathogens.

### Weak capacity of lectin-mediated recognition in mechanical trapping

Lectins, which are carbohydrate-binding proteins, are highly specific for their sugar moieties [37], and they are thought to be involved in fungus–nematode recognition, especially in adhesive trapping [38]. Lectin-mediated recognition has been identified in nematode-trapping fungi based on the presence of lectins associated with glycosyls of N-acetyl-D-galactosamine (GalNAc), D-glucose, D-mannose, and L-fucose [38-40] in different adhesive network-forming species. Seventeen lectin genes were predicted in *A. oligospora*. They mainly recognized GalNAc and fucose, e.g., four H-type lectins and three ricin B lectins recognized GalNAc, and four lectins recognized fucose (Additional file 2: Table S2). Consistent with the lectins identified from the surface of *A. oligospora* trapping organs [38], the presence of these GalNAc-binding lectin genes highlighted the role of GalNAc-recognition in adhesive trapping. However, the fucose-binding lectins do not match the lectins identified from traps and they should be further investigated.

Compared with *A. oligospora*, there were fewer lectin genes in the *D. stenobrocha* genome; only one GalNAc-binding lectin (4 in *A. oligospora*) and one fucose-binding lectin (5 in *A. oligospora*) were predicted (Additional file 2: Table S2), suggesting that *D. stenobrocha* had a weak capacity for lectin-mediated recognition. Given the mechanical-trapping mechanism of *D. stenobrocha*, lectin-mediated recognition might be functionally replaced by the actively ring constricting within a fraction of second (0.1 s). In addition, *D. stenobrocha* has a number of bulb-type lectins (Additional file 2: Table S2, Table S3), which are known to be involved in the defense of the cotton leafworm [41]. These facts suggest that *D. stenobrocha* may use lectins as a defense against nematodes rather that to adhere to them.

Lectins with the highest amino acid similarity were not classified into the same protein family, indicating multiple origins and convergent evolution (Figure 4C). Glycosyls bound by the lectins may change during fungus–nematode coevolution. These evolutionary changes could be adaptations to the prey of the adhesive-trapping fungi, although the biological significance of this coevolution is not yet clear.

### Predation originated in carbon-rich environments as evidenced by GH families and the rampant expansion of CBM1 genes

Because nematode-trapping fungi were hypothesized to have evolved in response to carbohydrate-rich but nitrogen-poor environments [11], we examined their carbohydrate use in detail, and the numbers of carbohydrate-active enzymes (CAZymes) were analyzed for differences between nematode-trapping fungi and others [42]. Nematode-trapping fungi had abundant glycoside hydrolases (GHs) for cellulose degradation. A total of 147 GH genes in *D. stenobrocha* genome and 226 in *A. oligospora* were predicted, similar to the average number (181) in entomopathogenic fungi but fewer than in plant pathogenic (280) and saprophytic (232) fungi (Additional file 2: Table S4).

In filamentous fungi, cellulose is mainly degraded by endoglucanases and cellobiohydrolases in the cellulase families GH1, GH3, GH5, GH6, GH7, and GH61 [43-48]. The GH5 family, previously known as "cellulase family A" (http://www.cazypedia.org), were the most highly expanded GH family. Both *D. stenobrocha* (15) and *A. oligospora* (20) contained larger number genes from the GH5 family compared to other fungi except *A. nidulans* (16). The GH61 family was first identified in cultures of *Phanerochaete carnosa* performing oxidative attack of crystalline cellulose on wood [49]. The *A. oligospora* genome has the most GH61 genes (26), nearly twice as many as *D. stenobrocha* and nine times as many as entomopathogens (1.5). However, phytopathogenic fungi averaged 19 GH61s and saprophytic fungi averaged 12, indicating that the cellulose-degrading capacity of nematode-trapping fungi was more like that of phytopathogens or saprobes than entomopathogens. This may also be supported by the niches where enriched in the cellulose materials (e.g. leaves) for trapping fungi and where was less cellulose materials (stem of plants and phyloplane) for entomopathogens. In addition, the GH7 family was well represented in nematode-trapping fungi (6 in *A. oligospora*, 4 in *D. stenobrocha*) compared with other fungi (average 3). The GH74 family, which encodes xyloglucans/xyloglucan-oligosaccharides, are also presented better in nematode-trapping fungi (3 in *D. stenobrocha*, 4 in *A. oligospora*) than other compared fungi (average 2).

Phytopathogens in particular have additional GH3, GH11, and other GH families classified in the pathogen–host interaction (PHI) database. The two nematode-trapping fungi (averaged 8 GH3 and 2 GH11 genes) had fewer than in phytopathogenic fungi (averaged 20 GH3 and 4 GH11 genes). The contraction of these genes associated with plant-infection might explain why nematode-trapping fungi contain fewer total GHs but have a similar capacity for cellulose degradation.

**Figure 5:**
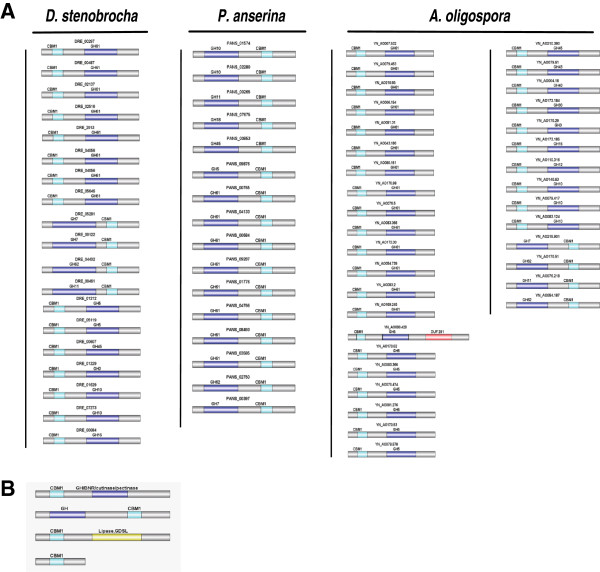
**CBM1 appended genes in *D. stenobrocha *genome compared with that of *A. oligospora *and *P. anserina. *A)** Glycoside hydrolases coding genes containing CBM1 domains in *D. stenobrocha* compared with that of *A. oligospora* and *P. anserina*, showing the expanded CBM1 appended glycoside hydrolases in nematode-trapping fungi and the different gene structures between nematode-trapping fungi and saprophytic fungi. **B)** Structures of CBM1 domain appended genes in *D. stenobrocha* genome. CBM1 + glycoside hydrolase; glycoside hydrolase + CBM1; CBM1 + lipase; CBM1.

The activity of CAZy enzymes can be enhanced by carbohydrate-binding module 1 (CBM1) protein domains within these proteins [50]. These domains may increase enzyme efficiency by enhancing the localization of enzymes on the surface of crystalline cellulose [50] or disturb the structure of crystalline cellulose [51]. There were 37 CBM1-containing genes in the *D. stenobrocha* genome, half the number (86) in *A. oligospora* but three to four times as many as in the other fungal genomes (average 12, Table 2). Twenty-six of the 37 *D. stenobrocha* CBM1-containing proteins had a signal peptide for secretion, indicating that these enzymes function extracellularly. The typical saprophytic white rot fungus (*Phanerochaete chrysosporium*) and the coprophilous *Podospora anserina* had the most CBM1-containing protein genes [52,53]. There were 19 GHs containing CBM1 domains in *D. stenobrocha* and 47 in *A. oligospora*, compared with 16 in *P. anserina* (Figure 5)*.* However, CBM1-containing polysaccharide lyases which were specific to plant pathogens and were absent in nematode-trapping and saprophytic fungi [25]. Thus, the CBM1 profiles of nematode-trapping fungi were similar to those of saprophytic fungi but different from those of plant pathogenic fungi.

The capacities of secreted enzymes to degrade carbohydrates and nitrogenous compounds might also be expected to differ among fungi, and this might be reflected in the genes related to their secretomes. Therefore, the secretomes of *D. stenobrocha* and *A. oligospora* were compared with those of entomopathogens to identify enzymes involved in carbon and nitrogen resource utilization. *A. oligospora* had a large number of secreted proteins (646) compared with entomopathogens (406 in *M. acridum*, 546 in *M. anisopliae*), and twice as many as *D. stenobrocha* (355) (Additional file 2: Table S5)*.* The GH proteins in the nematode-trapping fungi secretomes showed a completely different profile from those in entomopathogens. Carbohydrate use was enhanced in nematode-trapping fungi, while protein degradation was weakened. Indeed, carbohydrate-degrading enzymes were much more likely to be shared between nematode-trapping species (22% shared) than were proteases (5–6%), as opposed to in entomopathogens (9–10% and 12–14% shared, respectively) (Additional file 1: Figure S2). Thus the large number of predicted genes for carbohydrate-degradation and the reduced number for nitrogen resource use supported the hypothesis that nematode-trapping fungi originated in carbon-rich but nitrogen-poor environments.

### Transcriptional responses during nematode trap formation reveal clues to the transition from saprophagy to predation

To identify the putative signal and metabolic pathways involved in trap formation and the nematode trapping process, we used RNA-Seq to examine the transcriptional responses of *D. stenobrocha* during the nematode–fungus interaction (GEO accession: GSE54423; http://www.ncbi.nlm.nih.gov/geo). Three stages the interaction were examined: (1) *D. stenobrocha* cultures on CMA medium free of nematodes; (2) cultures with a large number of traps 22 h after nematode challenge; and (3) cultures in which most of the nematodes were trapped 28 h after nematode challenge [54]. After sequencing over 1 million tags for each treatment, 97.2% of the genes predicted in the *D. stenobrocha* genome were expressed in the cultures free of nematodes. After nematode challenge for 22 and 28 h, 27% of the predicted genes were differently expressed (probability ≥ 0.5, NOIseq; http://bioinfo.cipf.es/noiseq) compared to the nematode-free culture, including 800 that were up-regulated and 1,300 that were down-regulated.

**Figure 6:**
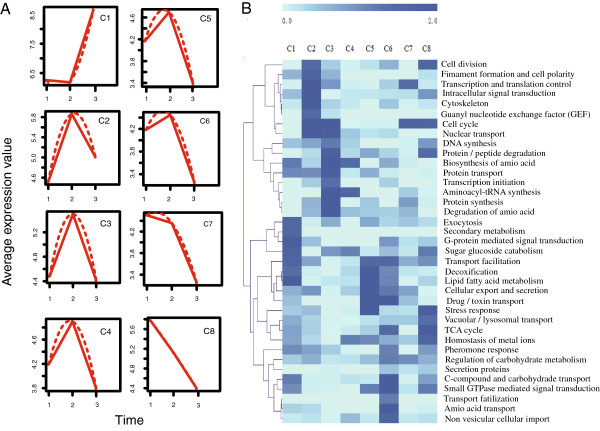
**Expression profiling of trap-formation and pathogen associated genes in *D. stenobrocha*. A)** Genes are profiled into 8 clusters as the expressing pattern, the three time points indicate the three samples for RNA-sequence; 1) Hyphae without nematode challenge; 2) Hyphae with 22-hour nematode challenge; 3) Hyphae with 28-hour nematode challenge. **B)** Heat-map of expressing genes shows the expressing pattern of different gene clusters, the annotation of these genes is based on FunCat database.

Transcripts were classified into eight functional clusters (maSigPro) to provide a global view of the transcriptional responses to nematode stimulation and trapping (Figure 6a). In general, the most significantly up-regulated genes were involved in rapid cell growth, intracellular signal transduction, and protein degradation, concomitant with the formation of trapping organs (Figure 6b). Of the 100 most highly-expressed genes during the development of the constricting rings (22 h), 35 were functionally uncharacterized (Additional file 2: Table S6), suggesting that several genes with unknown function were involved in the development of trapping organs. Additionally, 27 of these 35 genes were down-regulated to very low levels 28 h after nematode challenge (Additional file 2: Table S6). These genes should be identified to provide insight into trap formation.

Although the predatory lifestyle and constricting rings are unique to *D. stenobrocha*, during nematode trapping, signal transduction pathways activated were similar to those employed by entomopathogens during insect infection. For instance, the protein kinase C (PKC) signal transduction pathway is important for fungal infection of insects [23], and the up-regulation of protein kinase C (DRE_04067) also suggest a role in nematode trapping. In addition, the highly expressed G-protein alpha subunit (DRE_07451) might be the first step in the PKC pathway, activated by a protein signal from nematodes (Additional file 2: Table S7). Unexpectedly, pheromone receptors involved in fungal fruiting body formation and the sexual phase of the cycle [55] have been reported to be involved in nematode trapping in *A. oligospora*, but their orthologs (DRE_04115, DRE_03014 ) in *D. stenobrocha* were not transcribed or were transcribed at low levels during the capture of nematodes (Additional file 2: Table S7). Taken together, these data on pheromone receptor and signal transduction genes suggested that trap-formation of *D. stenobrocha* was more dependent on the PKC pathway than the pheromone-dependent signal pathway.

Following PKC-mediated signal transduction, transcription factors were activated to regulate the downstream genetic responses. Zn(2)–C6 type transcription factors were predicted to be involved in the regulation of aspects of both primary and secondary metabolism in *Aspergillum,* including protein and carbohydrate degradation [56]. The levels of these transcription factors, coincident with the up-regulation of proteases are predicted to be involved in the transition in *D. stenobrocha* from a saprophagous to a predatory stage.

The formation of constricting ring was expected to be significantly involved in the cell division. A cell division protein (DRE_05065) and a cyclin (DRE_07217) were highly expressed during the trap-formation (Additional file 2: Table S7). This indicates that these two cycle proteins may be the key genes that initiate the cell division for trap-formation. Furthermore, a large number of transporters, including six genes belong to MFS (major facility superfamily) among the 100 most highly up-regulated genes, were employed by *D. stenobrocha* to utilize the nutrition of nematodes (Additional file 2: Table S6).

Differential expression of secreted enzymes revealed the distinct transition from saprophagy to predation in *D. stenobrocha* during the nematode-trapping process. The down-regulation of enzymes involved in carbohydrate degradation, including cellulases (GH5, GH7, and GH61) and endoxylanase (GH10), indicated the reduced importance of carbohydrate use from the substrate during nematode trapping. In contrast, the secreted enzymes involved in cuticle degradation and infection, such as chitinases (GH18) and subtilisins (peptidase S8A), were up-regulated, suggesting a shift to predation. In addition, other putative serine proteases involved in nematode infection were significantly up-regulated (Table S8). These results support the hypothesis that nematode-trapping fungi are both efficient carbohydrate utilizing saprobes and nematode-trapping predators. In addition, other putative serine proteases (peptidases S28 and S33) involved in nematode infection were significantly up-regulated. The transcriptional profiles revealed well the transition from saprophagy to predation in nematode-trapping fungi.

## Discussion

Fungal predation, especially the evolutionary origins of nematode trapping fungi and their mechanical trapping mechanism, have long attracted the interest of mycologists and other biologists. The three cells of the constricting ring rapidly triple their volumes to firmly lasso their nematode prey, but this fascinating biological phenomenon is still poorly understood. This analysis of the genome and transcriptome of *D. stenobrocha* in comparison with the genome of the adhesive network-forming *A. oligospora* provides insights into the origins of fungal predation, the shift from a saprophagous to a predatory stage, and molecular mechanisms of nematode trapping.

Whole genomic sequences indicated that *D. stenobrocha* is compact, showing rare RIPs and transposons. The gene NCU02034 in *N. crassa*, which is only known to be required for RIP [27], was absent from the *D. stenobrocha* genome, indicating that it lacks the ability for RIPs. The rate of gene duplication is decreased by RIP [57], implying that genomes with high frequency of RIPs generally have low gene duplication rates. *D. stenobrocha* is an exception. The paucity of RIP and transposons in the *D. stenobrocha* genome indicated that it was compact with fewer orphan genes arose compared with that of *A. oligospora*; only 7,781 genes were predicted. Given the function of RIPs in meiosis [27], more studies should be conducted to investigate whether *A. oligospora* or *D. stenobrocha* undergo sexual stages.

Previous studies showed that fungal recognition of the nematode was mediated by lectins on the trap surface [38]. Thus far, six kinds of lectins that recognize different glycosyls have been identified; those glycosyls are GalNAc in *A. oligospora*, D-glucose in *Arthrobotrys conoides*, L-fucose in *Monacrosporium eudermatum*, 2-deoxy-D-glucose (2-DG) in *Monacrosporium rutgerienses*, sialic acid in *Drechmeria coniospora*, and N-acetylneuraminic in *Verticillium balanoides*[38-40,58]. The presence of lectins that bind GalNAc and L-fucose in *A. oligospora* partially supported the role of lectins in host–pathogen recognition. However, the presence of fewer lectins in the *D. stenobrocha* genome suggested that the mechanical-trapping mechanism might not involve lectin in the fungal recognition of nematodes. Adhesive proteins are involved in host recognition by plant and insect fungal pathogens [33,34]. The occurrence of adhesive proteins in two species of trapping fungi suggested that they may also play crucial roles in both mechanical and adhesive trapping.

Plant residues in forests generally result in an extremely high carbon-to-nitrogen ratio. Some wood-decaying fungi may have evolved to capture tiny animals for nitrogen under this environmental selection pressure [11]. A phylogenetic analysis based on the conservative genes of nematode-trapping fungi belonging to Ascomycetes also suggests a possible causal relationship between mass extinction events and the evolution of fungal predation [9]. Predatory fungi gained a competitive advantage over strict saprobes by predating tiny animals when available organic matter decreased during the ecosystem recovery [9]. Although this hypothesis lacks direct evidence, comparative analyses of the genome of *D. stenobrocha* provide support for this hypothesis.

In this study, comparison of the selected genomes revealed that genes involved in cellulolytic degradation were abundant in nematode-trapping fungi, whereas genes for enzymes that degrade living plant cells were rare, indicating that nematode-trapping fungi share more similarities with saprobes than with phytopathogens. The divergence time of the adhesive-trapping fungus, *A. oligospora* with greater saprophytic capacity, was much later than that of *D. stenobrocha*[9]. Our hypothesis that nematode-trapping fungi originated from efficient cellulolytic fungi was partially supported by evidence that constricting ring-trapping fungi with weaker saprophytic capacity evolved to capture nematodes after the mass extinction events of the Permian–Triassic (251.4 MY), whereas adhesive-trapping fungi survived the Permian–Triassic boundary with the enhanced saprophytic capacity and evolved to capture nematodes after the mass extinction of the Triassic–Jurassic. More genomes of nematode-trapping fungi with different trapping organs should be analyzed.

The density dependence of nematophagous fungi also varies with the availability of saprophytic component. Adhesive net-forming *A. oligospora* has higher diversity in the nutrition poor niches indicating its higher saprophytic capacity [14]; while constricting ring-forming *D. stenobrocha* always exists in the nutrition rich niches suggesting its weaker saprophytic capacity than *A. oligospora*[14]. The enhanced saprophytic capacity of *A. oligospora* was evidenced by the greater expansion of CBM1-containing GHs, which revealed that *A. oligospora* depends more on saprophagy than does *D. stenobrocha*, suggesting that *A. oligospora* is more competitive when nematodes are absent, in addition *D. stenobrocha* might be more dependent on nematodes for nutrition. The dependence of *D. stenobrocha* on nematodes might explain why its constricting rings can form spontaneously, whereas the network of *A. oligospora* must always be induced.

Although the genome sequence of *D. stenobrocha* does not provide specific evidence about ring-cell inflation nor ring constriction, a number of orphan genes in the genome may contribute specifically to this process. Among the 470 expressed orphan genes, more than 120 were differently expressed in the presence of nematodes, suggesting that they encode proteins that are involved in ring constriction. However, more than 90 of these orphan genes were not annotated in the Pfam database, including a large number of those that were most significantly up-regulated. These uncharacterized genes that were expressed during ring constriction imply that a novel mechanism is involved. These genes provide candidates to investigate in future functional studies.

## Conclusion

In conclusion, the rare occurrence of transposons and RIPs indicated the slow evolution and primitive state of the *D. stenobrocha* genome. The similarity of carbohydrate-degrading enzyme catalogs between trapping fungi and efficient cellulolytic fungi support the saprophytic origin of predation. The down-regulation of saprophagy-related genes and the up-regulation of predation-related ones during nematode infection revealed the transition between dual strategies of saprophagy and predation in this trapping fungus. The reduced number of lectin genes in *D. stenobrocha* did not support lectin-mediated recognition in mechanical trapping. The high expression levels of uncharacterized orphan genes imply that an unknown mechanism is involved in ring constriction. Overall, the genomic and transcriptome sequences of *D. stenobrocha* provided new insight into the origin of nematode predation in fungi and the mechanism of constricting rings, these sequences also can be essential tools to reveal the mechanism of nematode-capture by constricting rings, and for further better exploitation of nematode-trapping fungi in the use of nematode control.

## Methods

### Fungal strains

*D. stenobrocha* strain (CGMCC 3.6768) was selected for genome sequencing and has been studied well in the laboratory which can be cultured on artificial medium such as PDA and CMA plates and maintained on PDA Plate. *D. stenobrocha* spores were cultured on CMA plate for 4 days and challenged with 500 nematodes each plate. Samples were prepared [59] and structures were observed by scanning electron microscope Quanta200, produced by FEI. This fungus was originally isolated by Drechsler in 1937 and we isolated from the soil of Yunnan province in 2004. It provides important materials to research the mechanisms of constricting rings.

### Genome sequencing and assembly

The genome of *D. stenobrocha* was sequenced by shotgun using a Solexa system for massively parallel pyrosequencing at BGI (Shenzhen, China). This resulted in 4,299 Mb of sequence data (80 × coverage) with the useful length of 2,317 Mb. By using the SOAPdenovo software [60], assembly was performed producing 142 contigs and reached the size of 29.02 Mb.

### Gene prediction and annotation

In order to reach high accuracy, the gene structures of *D. stenobrocha* were predicted with EVidenceModeler (EVM) [61] algorithms and the sequenced *Fusarium graminearum* was used as a reference. PseudoPipe was selected with default settings conducting the pseudogene identification [62]. Finally, the prediction was performed by Blast against protein database and InterProscan searches against protein domain databases. Potential secreted proteins of *D. stenobrocha* and other species were predicted and compared by SignalP 3.0 [63] analysis using Hidden Markov model.

### Orthology and phylogenomic analysis

Predicted proteins of *D. stenobrocha* were compared with the predicted proteins of other sequenced 9 fungi. The comparison of all proteins was performed by BLASTP against all the other proteins in these genomes. Sequences with E ≤ 1e^-5^ and as least 40% sequence identity over 60% were considered homologous sequences. Totally 1441 ortholog genes were acquired and aligned with MAFFT [64]. Amino acid sequences were used by the program RAxML to create a maximum likelihood tree [65]. Divergence time between the compared species was estimated by PL method [66] with r8s version 1.8 (http://loco.biosci.arizona.edu/r8s/) using the calibration against the origin of Ascomycota at 500–600 million years ago [67].

### Protein family classification and evolution analysis

Protein families of the whole genome were classified by use of InterproScan analysis in order to identify genes descended from a common ancestor [68]. Putative enzymes involved in carbohydrate utilization were identified by blast searching against carbohydrate-active enzymes database (http://www.cazy.org/). Protease protein families were classified by blast against MEROPS database. And additionally, G-protein coupled receptors, protein kinases, transcription factors were identified by the significant sequence of GPCDB 7 transmembrane helices (http://www.cbs.dtu.dk/services/TMHMM/), KinBase (http://kinase.com/) and fungal transcription database (http://ftfd.snu.ac.kr/). The evolution of the protein families’ size and knot point was analyzed by CAFÉ [69].

### Repeat and Repeat-induced point mutation (RIP) analysis

Genomic repetitive analyses were performed by Blasted against the PILER [70], PepeatMasker library [71] and TRF [72]. RIP index was determined by program RIPCAL [26].

### Transcriptome analysis

*Caenorhabditis elegans* were cultured in NGM manual liquid medium for 4 days, and collected by 0.01 mm filter membrane. The *Escherichia coli* strain, OP50, was cultured in LB medium for food of nematodes [73]. The spores of *D. stenobrocha* with the number of 5.2 × 10^5^ were cultured in 100-mL PDB liquid medium for four days. Hyphae was collected by glass cotton felt and washed with water three times. Hyphae from each 100-mL liquid medium were left on two CMA medium plate for two days and co-cultured with 2500 *C. elegans* each plate. After 22- and 28-hours challenge at 25°C, RNA and was exacted by the method of TRizol [74]. Functions of the expressed genes were predicted based on FunCat database. Program maSigPro was performed to enrich the genes expressed in different patterns [75].Probability value of each differently expressed gene is calculated by program NOISeq [76].

## Abbreviations

2-DG: 2-deoxy-D-glucose; cAMP: Cyclic adenosine monophosphate; CAZyme: Carbohydrate-active enzymes; CBM1: Carbohydrate-binding module 1; CDC: Cell division cycle; GalNAc: N-acetyl-D-galactosamine; MFS: Major facility superfamily; MY: Million years; GH: Glycoside hydrolase; IP3: Inositol-1, 4, 5-triphosphate; PHI: Pathogen–host interaction; PKC: Protein kinase C; RIP: Repeat-induced point.

## Competing interests

The authors declare that they have no competing interests.

## Authors’ contributions

KL conducted the molecular experiments. XZ assembled the genome and annotated the genes. WZ analyzed the data and wrote the manuscript. YL and XW helped to analyze the data. MX helped with the molecular experiments. XL designed the study and wrote the paper. All authors have read and approved the manuscript.

## Supplementary Material

Additional file 1: Figure S1 Distribution of paralogous gene numbers with different levels of nucleotide similarity in *D. stenobrocha* and other fungi. **Figure S2.** Classification and comparison of secreted proteins by function of nematode-trapping fungi (*D. stenobrocha*, *A. oligospora*) and insect pathogens (*M. acridum*, *M. anisopliae*). Each circle represents fraction of genes in each genome, and the percentages are also shown. Click here for file

Additional file 2: Table S1 Genome sizes and numbers of protein coding genes in *Drechslerella stenobrocha* and other fungi. **Table S2.** Abundance of lectins and adhesive proteins in *Drechslerella stenobrocha*, *Arthrobotrys oligospora*, and other fungi. **Table S3.** Predicted lectin coding genes in *Drechslerella stenobrocha* and *Arthrobotrys oligospora* genomes. **Table S4.** Carbohydrate-degrading enzymes in *Drechslerella stenobrocha* and other fungi arranged by glycoside hydrolase family. **Table S5.** Numbers of secreted proteins in the *Drechslerella stenobrocha* and other fungal genomes. **Table S6.** The 100 most differentially-expressed genes in *Drechslerella stenobrocha* while trapping nematodes. **Table S7.** Transcriptional response of genes involved in signal transduction of *Drechslerella stenobrocha*. **Table S8.** Expression of secreted enzymes in the three transcriptomes of *Drechslerella stenobrocha*. Click here for file
